# Adjuvant chemotherapy, extent of resection, and immunoistochemical neuroendocrine markers as prognostic factors of early‐stage large‐cell neuroendocrine carcinoma

**DOI:** 10.1111/1759-7714.14287

**Published:** 2022-02-16

**Authors:** Claudio Andreetti, Mohsen Ibrahim, Antonio Gagliardi, Camilla Poggi, Giulio Maurizi, Domenico Armillotta, Valentina Peritone, Leonardo Teodonio, Erino Angelo Rendina, Federico Venuta, Marco Anile, Giovanni Natale, Mario Santini, Alfonso Fiorelli

**Affiliations:** ^1^ Thoracic Surgery Unit, Sant'Andrea Hospital University of Rome La Sapienza Rome Italy; ^2^ Thoracic Surgery Unit, Policlinico Hospital University of Rome La Sapienza Rome Italy; ^3^ Thoracic Surgery Unit University of Campania Luigi Vanvitelli Naples Italy

**Keywords:** adjuvant chemotherapy, immunoistochemical neuroendocrine markers, large‐cell neuroendocrine carcinoma, lobectomy, surgery

## Abstract

**Background:**

We investigated whether adjuvant chemotherapy, extent of resection, and immunoistochemical neuroendocrine markers affected survival of patients with the early stage of large‐cell neuroendocrine cancer.

**Methods:**

This was a retrospective multicenter study including consecutive patients undergoing resection of node negative large‐cell neuroendocrine carcinoma. Five‐year survival and disease‐free survival rate were evaluated by the Kaplan–Meier method and the log‐rank test in relation to adjuvant chemotherapy, extent of resection, and immunoistochemical neuroendocrine markers (synaptophysin, chromogranin A, and neuron‐specific enolase).

**Results:**

Our study population included 117 patients; 47 (40%) of these received adjuvant chemotherapy. Patients treated with adjuvant chemotherapy had better survival (74% vs. 45%, *p* = 0.002) and disease‐free survival (79% vs. 40%, *p* = 0.001) in all cases except patients with tumor <20 mm (79.5% vs. 57.4%, *p* = 0.43). Lobectomy compared to sublobar resection was associated with better survival (67% vs. 0.1%, *p* < 0.0001) and disease‐free survival (65% vs. 0.1%, *p* < 0.0001) also in patients with tumor <20 mm (79% vs. 28%, *p* = 0.001). Patients with triple‐positive neuroendocrine markers had better survival (79% vs. 35%, *p* = 0.0001) and disease‐free survival (69% vs. 42%, *p* = 0.0008). Regression analysis showed that tumor size <20 mm, lobectomy, adjuvant chemotherapy, and triple‐positive immunistochemical neuroendocrine markers were significant favorable prognostic factors for survival outcomes.

**Conclusions:**

Lobectomy seems to be the management of choice in patients with large‐cell neuroendocrine cancer <20 mm while adjuvant chemotherapy should be administered only in patients with tumor >20 mm.

## Graphical abstract

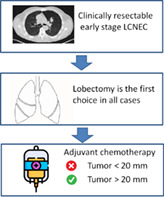

## INTRODUCTION

Large‐cell neuroendocrine carcinoma (LCNEC) of the lung is a rare malignant tumor and accounts for only 2–3% of all primary lung cancers.^1^ Although previously classified as a subgroup of large‐cell carcinoma, in 2015 LCNEC was reclassified as a high‐grade neuroendocrine tumor, including the subgroups small‐cell lung cancer (SCLC), typical carcinoid, and atypical carcinoid.^2^

Primary surgery remains the main treatment for patients with limited LCNEC, but the prognosis is poor even in patients with pathologic stage I because of its aggressive course and high potential for metastasis of LCNEC. This led many physicians to consider LCNEC together with SCLC and to routinely do adjuvant chemotherapy regardless of pathologic stage, while others did not show any benefits associated with adjuvant chemotherapy even in early stage of LCNEC.^1^, ^3^ The different clinico‐pathological features of LCNEC among these studies likely explain the contrasting results. Thus, the optimal treatment remains to be established in these subsets of patients.

In this study, we investigated clinico‐pathologic features and survival outcomes in patients with early‐stage LCNEC to evaluate whether adjuvant chemotherapy, extent of resection, and immunoistochemical neuroendocrine markers affect survival outcomes.

## MATERIALS AND METHODS

### Study design

This was a retrospective multicenter study including the clinical data of consecutive patients undergoing intent‐curative surgery and receiving a diagnosis of LCNEC in three different thoracic surgery centers from January 2010 to January 2020. We excluded (i) patients with incomplete data sets and follow‐up, (ii) patients with lymph node involvement (>N0) and/or metastatic disease (M1), (iii) patients with tumor larger than 50 mm, (iv) patients with margin‐positive resection (R1, R2, or unknown), (v) patients with a diagnosis of mixed LCNEC combined with elements of SCLC, (vi) patients who died within 30 days of surgery; and (vii) patients without immunistochemical investigation of the following neuroendocrine markers: synaptophysin, chromogranin A, and neuron‐specific enolase (NSE).

The end points of the paper were to evaluate the impact of adjuvant chemotherapy, type of resection, and immunoistochemical neuroendocrine markers on survival outcomes (overall survival and disease‐free survival) in order to stratify the best treatment for each subgroups of patients.

The study protocol was approved by the local ethics committees of each participating center; no specific code approval was needed because it was a retrospective study that did not change the standard clinical practice. All patients gave written informed consent for the treatment and the data were anonymously used.

### Patients' data

The following parameters were investigated from the medical records: patient gender, age, smoking index, pathologic tumor size, surgical procedure, regimen of adjuvant chemotherapy, time and site of recurrence, and time and cause of death. Before operation all patients underwent standard cardio‐respiratory evaluation and oncological staging through imaging of the chest and abdomen, and positron emission tomography (PET). If indicated, histopathology evaluation of mediastinal nodes was performed via cervical mediastinoscopy, endobronchial ultrasound (EBUS)‐guided transbronchial needle aspiration (TBNA), thoracoscopy, or chamberlain incision. Resections included lobectomies and sublobar resections (segmentectomy or wedge resection) as indicated. Systematic lymph node dissection was carried out in all cases. The diagnosis of LCNEC was confirmed based on the WHO criteria, including the presence of neuroendocrine morphology and positive staining for synaptophysin, chromogranin A, and NSE.^4^ Tumors were categorized as pure LCNEC or combined LCNEC based on the presence of mixed histologic components such as adenocarcinoma, squamous cell carcinoma, or giant cell carcinoma. Based on tumor size, patients were divided into three groups: tumors <20 mm, tumors between 20 and 30 mm, and tumor between 30 and 50 mm.

### Postoperative treatment and follow‐up

Adjuvant chemotherapy was defined as given after the surgical resection while treatment given for disease progression or recurrence was excluded. Chemotherapeutic regimen, time of administration from surgery, and duration of treatment were at the discretion of the treating centers. Five‐year survival rate (5‐YSR) was calculated from the day of operation to the date of death from any cause or of the last follow‐up. Five‐year disease‐free survival rate (5‐YDFSR) was calculated from the day of operation to the time of the first recurrence. Tumor recurrence and cause of death were determined for each patient. Loco‐regional recurrence was defined as that occurring within the ipsilateral hemithorax while distant recurrence was defined as that developing within the contralateral hemithorax or a distant solid organ.

### Statistical analysis

The summary statistics of patient characteristics were tabulated either as mean ± standard deviation (SD) for continuous variables or as number of patients and percentages for categorical variables. Student's *t* test and the chi‐square test were used to compare different variables, as appropriate. The 5‐YSR and the 5‐YDFSR were evaluated by the Kaplan–Meier method and the log‐rank test was used to calculate the difference between subgroups. The Cox multivariate proportional hazards regression model was used to identify independent risk factors for death and recurrence. A *p* value of less than 0.05 was considered statistically significant. MedCalc statistical software (version 12.3) was used.

## RESULTS

In the study period, 153 patients underwent surgical resection for LCNEC. Among these, 36 patients were excluded because of missing clinical data and incomplete follow‐up (*n* = 13), of N1‐N2 disease (*n* = 7), of tumor larger than 50 mm (*n* = 10), of diagnosis of mixed LCNEC combined with elements of SCLC (*n* = 3), or of lack of investigation of immunistochemical neuroendocrine markers (*n* = 3). Thus, our study population included the 117 patients summarized in Table 1.

**TABLE 1:** Study population

Variable	All	Adjuvant chemotherapy	No adjuvant chemotherapy	*p* value
Number of patients	117	47 (40%)	70 (60%)	–
Age (years)	67 ± 3.9	67 ± 9.8	67 ± 7.9	0.83
Sex (male)	87 (74%)	37 (79%)	50 (71%)	0.37
Smokers	110 (94%)	42 (89%)	68 (97%)	0.08
Previous comorbidity (total)	91 (78%)	35 (78%)	56 (80%)	0.48
Diabetes	15 (65%)	7 (20%)	8 (14%)	
Hypertension	15 (65%)	5 (14%)	10 (18%)	
Cardiac	21 (31%)	8 (23%)	13 (23%)	
Cerebral	1 (1%)	0	1 (2%)	
COPD	30 (33%)	11 (31%)	19 (34%)	
Neoplastic	9 (10%)	4 (12%)	5 (9%)	
Symptoms				
None	50 (27%)	21 (47%)	29 (41%)	0.72
Cough	25 (21%)	17 (36%)	18 (26%)	
Thoracalgia	5 (4%)	2 (4%)	3 (4%)	
Expectoration	6 (5%)	2 (4%)	4 (6%)	
Hemoptysis	9 (8%)	4 (8%)	5 (7%)	
Pyrexia	7 (6%)	3 (6%)	4 (6%)	
Weight loss	15 (13%)	6 (13%)	9 (13%)	
Respiratory function				
FEV1%	78 ± 21	78 ± 15	77 ± 32	0.45
DLCO %	73 ± 18	73 ± 22	72 ± 21	0.21
6‐MWT (metres)	365 ± 59	366 ± 63	364 ± 49	0.39
Tumor site				
Peripheral	79 (67%)	30 (64%)	49 (70%)	0.38
Central	38 (23%)	17 (36%)	21 (30%)	
PET				
Mean value SUV value	6.9 ± 2.9	6.7 ± 2.9	6.9 ± 4.9	0.29
Patients with SUV > 2.5	113 (%)	45 (96%)	68 (97%)	0.68
Preoperative biopsy (total)	85 (73%)	35 (74%)	50 (71%)	0.71
Diagnostic	8 (9%)	3 (6%)	5 (7%)	
Inconclusive	5 (6%)	2 (4%)	3 (4%)	
Positive for malignancy	72 (85%)	32 (68%)	40 (57%)	
Type of resection				
Lobectomy	97 (83%)	40 (85%)	57 (81%)	0.60
Segmentectomy	17 (14%)	6 (12%)	11 (16%)	
Wedge resection	3 (3%)	1 (3%)	2 (3%)	
Complications (total)	21 (4%)	4 (8%)	17 (24%)	0.03
Prolonged air leak	11 (52%)	1 (2%)	10 (14%)	
Atelectasis	3 (14%)	3 (6%)	0	
Atrial fibrillation	6 (28%)	0	6 (8%)	
ARDS	1 (6%)	0	1 (1%)	
Histology				
Pure	90 (77%)	35 (74%)	55 (78%)	0.62
Mixed	27 (23%)	12 (26%)	15 (22%)	
pTumor size	3.9 ± 2.5	3.8 ± 1.1	3.9 ± 1.5	0.49
<20 mm	29 (25%)	12 (25%)	17 (24%)	0.87
20 to 30 mm	46 (40%)	19 (40%)	27 (38%)	0.84
30 to 50 mm	42 (35%)	16 (35%)	26 (38%)	0.73
Immunoistochemical neuroendocrine markers				
Triple positive	67 (57%)	13 (32%)	54 (77%)	<0.0001
Nontriple‐positive	50 (43%)	34 (68%)	16 (23%)	<0.0001
Recurrence (total)	45 (38%)	10 (21%)	35 (50%)	<0.0001
Loco‐regional	30 (67%)	7 (15%)	23 (33%)	
Distant	5 (11%)	2 (4%)	3 (4%)	
Loco‐regional + distal	10 (22%)	1 (2%)	9 (13%)	

*Abbreviations*: 6‐MWT, 6‐minute walking test; ARDS, acute respiratory distress syndrome; COPD, chronic obstructive pulmonary disease; DLCO, diffusing capacity of the lungs for carbon monoxide; FEV1%, forced expiratory volume in the first second; SUV, standard uptake value.

The mean age was 67 ± 3.9 years old. All patients but seven (94%) were current smokers or had a history of intense tobacco consumption. At the time of presentation, 50 (27%) patients were asymptomatic and the tumor was found incidentally on a chest computed tomography (CT) scan. There were 79 (67%) peripheral and 38 (23%) central tumors shown by CT imaging and in all cases CT did not show specific features that were meaningful for the differential diagnosis of other types of lung cancer. A preoperative biopsy was made in 85 patients (73%). Only a small fraction of these patients were diagnosed with LCNEC (*n* = 8, 9%), while most were diagnosed with nonspecific cell types, including NSCLC (*n* = 72, 85%). In the remaining 32 (27%) patients intraoperative biopsy was performed with diagnosis of NSCLC in 30 patients and of LCNEC in two. Operative procedures performed included 97 lobectomies (84.1%) and 20 sublobar resections (17 segmentectomies and three wedge resections). LCNEC was categorized as pure (*n* = 90, 77%) or mixed (*n* = 27, 23%) with features of both LCNEC and NSCLC, mainly adenocarcinoma (73%). The mean tumor size was 3.9 ± 2.5 cm; 29 (25%) patients had tumor less than 20 mm, 46 (40%) tumor >2 to 30 mm, and 42 (35%) tumor >30 to 50 mm. Twenty‐one (4%) patients had postoperative complications including prolonged air‐leaks (*n* = 11, 52%), atelectasis (*n* = 3, 14%), atrial fibrillation (*n* = 6, 28%), and acute respiratory distress syndrome (ARDS) (*n* = 1, 6%).

### Recurrence and survival

Forty‐five (38%) patients had recurrence, 30 (67%) local recurrence, five (11%) distant recurrence, and 10 (22%) developed both local and distant recurrences. A total of 38 out of 45 (84%) patients were treated for recurrence with chemotherapy (*n* = 25, 65%), radiotherapy (*n* = 5, 13%), and combined radio‐chemotherapy (*n* = 8, 22%). The median follow‐up was 41 months (range 10–130 months). At the end of follow‐up, there were 75 surviving patients (64%); 11 (15%) of whom had progressive disease. Forty‐two (36%) patients died from disease progression (*n* = 34, 81%), cardiac disease (*n* = 5, 12%), and respiratory failure (*n* = 3, 7%). The 5‐YSR and 5‐YDFSR were 53% and 52%, respectively.

### Survival in relation to adjuvant chemotherapy

**FIGURE 1:**
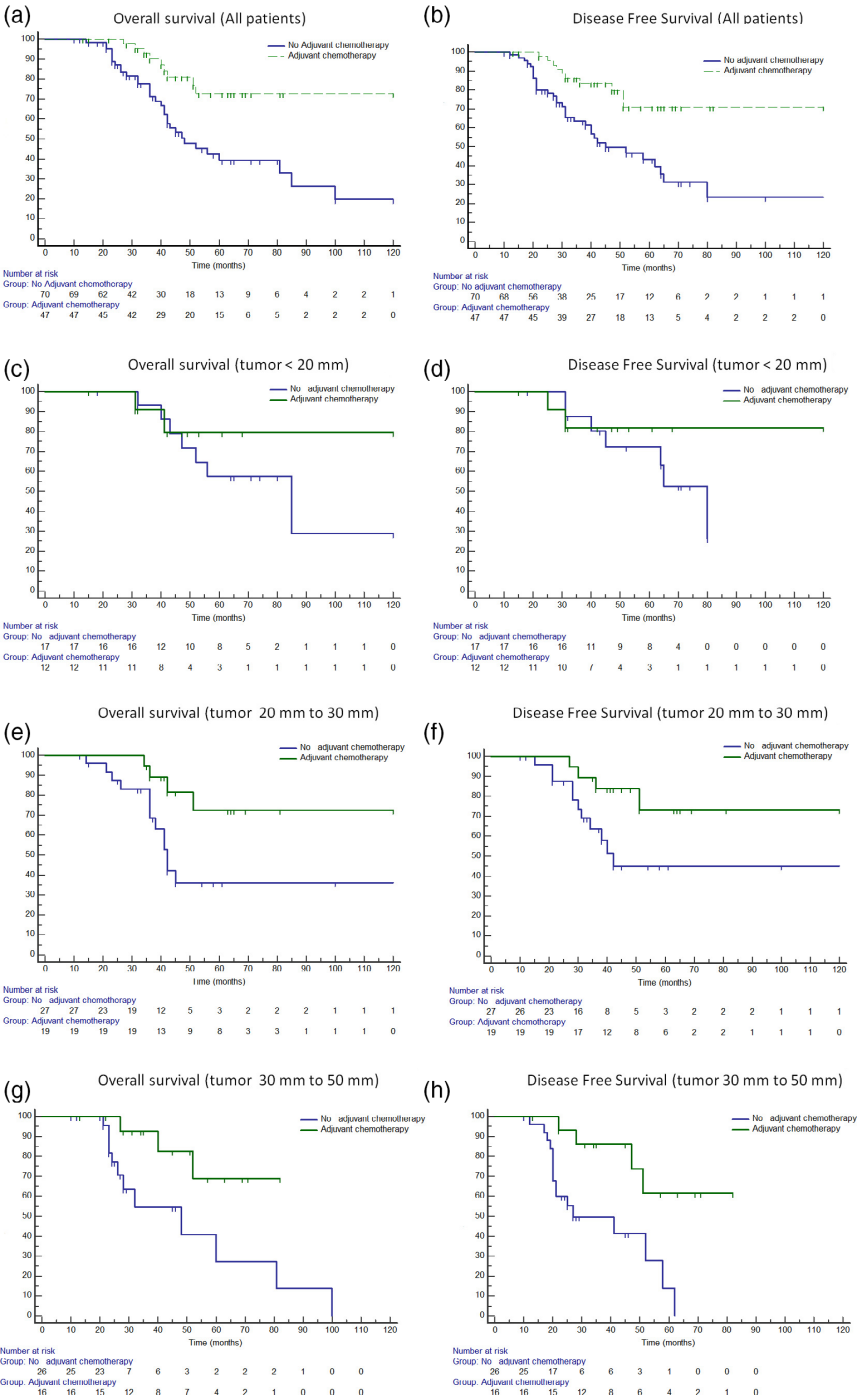
Five‐year survival and disease‐free survival rate in relation to administration of adjuvant chemotherapy in all patients (74% vs. 45%, *p* = 0.002 (a) and 79% vs. 40%, *p* = 0.001 (b)), in patients with tumor <20 mm (79.5% vs. 57.4%, *p* = 0.43 (c) and 81% vs. 72%, *p* = 0.51 (d)), in patients with tumor between 20 and 30 mm (72% vs. 36%, *p* = 0.01 (e) and 71% vs. 43%, *p* = 0.02 (f)), and in those with tumor >30 to 50 mm (68.8% vs. 27%, *p* = 0.01 (g) and 61% vs. 13.8%, *p* = 0.002 (h))

The results are summarized in Tables 1 and 2. Seventy (60%) patients underwent surgical resection alone and 47 (40%) received adjuvant chemotherapy. These patients were treated with an SCLC‐based regimen (etoposide/cisplatin, *n* = 20, 42%) or an NSCLC‐based regimen (*n* = 27, 58%) including gemcitabine (*n* = 7, 26%), vinorelbine (*n* = 7, 26%), pemetrexed (*n* = 7, 26%), and taxol (*n* = 6, 22%). Patients with less postoperative complications (*p* = 0.03) and no triple‐positive immunistochemical neuroendocrine markers (*p* < 0.0001) were more likely to receive chemotherapy after surgery. Patients treated with adjuvant chemotherapy compared to those who did not receive adjuvant chemotherapy had better 5‐YSRT (74% vs. 45%, *p* = 0.002; Figure 1(a)) and 5‐YDFSR (79% vs. 40%, *p* = 0.001; Figure 1(b)). When adjuvant chemotherapy was stratified in relation to tumor size, no significant differences were found in patients with tumor <20 mm (79.5% vs. 57.4%, *p* = 0.43, Figure 1(c); 81% vs. 72%, *p* = 0.51, Figure 1(d)) while adjuvant chemotherapy was associated with better 5‐YSR rates and 5‐YDFSR in patients with tumor >20 to 30 mm (72% vs. 36%, *p* = 0.01, Figure 1(e); 71% vs. 43%, *p* = 0.02, Figure 1(f)) and in those with tumor >30 to 50 mm (68.8% vs. 27%, *p* = 0.01, Figure 1(g); 61% vs. 13.8%, *p* = 0.002, Figure 1(h)).

**TABLE 2:** Survival and recurrence in relation to adjuvant chemotherapy, extent of resection, and immunoisthechemical neuroendocrine markers

Variables	Outcomes	Subgroups	Results	HR (95% CI)	*p* value
Adiuvant chemotherapy (*n* = 43) versus no adjuvant chemotherapy (*n* = 74)	5‐YSR	All patients	74% vs. 45%	2.8 (1.46–5.55)	0.002
		Tumor <20 mm	79.5% vs. 57.4%	1.7 (0.43–4.92)	0.43
		Tumor 20 to 30 mm	72% vs. 36.2%	3.2 (1.23–8.73)	0.01
		Tumor >30 to 50 mm	68.8% vs. 27%	3.4 (1.18–10.2)	0.01
	5‐DFSR	All patients	79% vs. 40%	2.81 (1.47–5.17)	0.001
		Tumor <20 mm	81% vs. 72%	1.7 (0.42–7.19)	0.51
		Tumor 20 to 30 mm	73% vs. 45%	2.8 (1.02–7.92)	0.02
		Tumor >30 to 50 mm	61% vs. 13.8%	4.0 (1.60–10)	0.002
Lobectomy vs. sublobar resection	5‐YSR	All patients	67% vs. 0%	11.6 (4.29–31.7)	<0.0001
		Tumor <20 mm	79% vs. 28.6%	8.2 (1.63–42.1)	0.001
		Tumor 20 to 30 mm	62% vs. 14.8%	13.5 (3.17–58.2)	0.0004
		Tumor >30 to 50 mm	51% vs. 0%	15.8(6.38–75.5)	<0.0001
	5‐YDFSR	All patients	65% vs. 0%	12 (4.57–31.8)	<0.0001
		Tumor <20 mm	89% vs. 38%	34 (5.91–189)	0.001
		Tumor 20 to 30 mm	71.5% vs. 0%	32.3 (6.52–160)	<0.0001
		Tumor >30 to 50 mm	30% vs. 0%	85 (7.85–175)	0.0001
Triple‐positive markers vs. no triple‐positive markers	5‐YSR	All patients	79% vs. 35%	3.92 (1.99–7.72)	0.0001
		Tumor <20 mm	92.3% vs. 36.9%	5.3 (1.32–21.6)	0.01
		Tumor 20 to 30 mm	74.9% vs. 28.1%	3.2 (1.21–8.72)	0.01
		Tumor >30 to 50 mm	60.4% vs. 29%	4.81(1.63–14.5)	0.0005
		Adjuvant chemotherapy	61% vs. 90%	0.23 (0.05–9.58)	0.043
		No adjuvant chemotherapy	72.3% vs. 16%	1.2 (1.95–9.40)	0.0003
	5‐YDFSR	All patients	69% vs. 42%	3.09 (1.60–5.97)	0.0008
		Tumor <20 mm	84% vs. 62%	2.53 (0.65–9.76)	0.03
		Tumor 20 to 30 mm	79% vs. 28%	3.15 (1.10–8.94)	0.03
		Tumor >30 to 50 mm	38% vs. 23%	4.11 (1.61–10.5)	0.003
		Adjuvant chemotherapy	68% vs. 91%	0.25 (0.05–1.13)	0.031
		No adjuvant chemotherapy	66% vs. 20%	3.83 (1.73–8.46)	0.0008

*Abbreviations*: 5‐YDFSR, 5‐year disease free survival rate; 5‐YSR, 5‐year survival rate.

### Survival in relation to extent of resection

**FIGURE 2:**
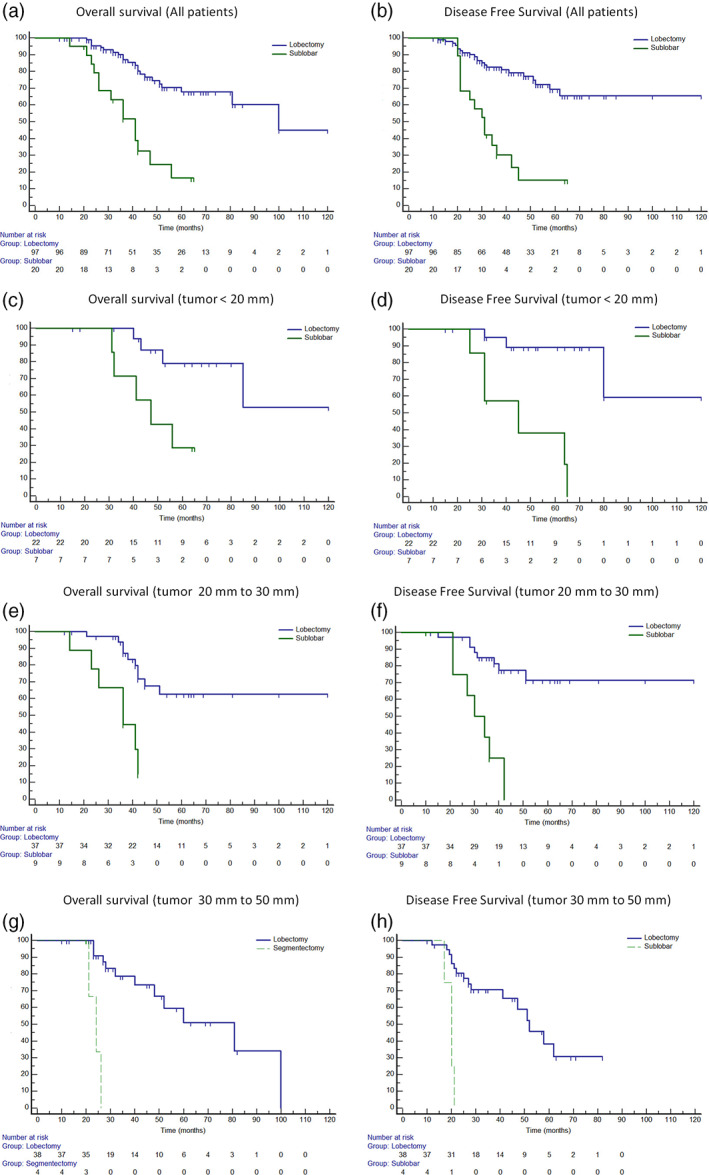
Five‐year survival and disease‐free survival rate in relation to type of resection (lobectomy vs. sublobar) in all patients (67% vs. 0%, *p* < 0.0001 (a) and 65% vs. 0%, *p* < 0.0001, (b)), in patients with tumor <20 mm (79% vs. 28%, *p* = 0.001 (c) and 89% vs. 38%, *p* = 0.001 (d)), with tumor between 20 and 30 mm (62% vs. 14.8%, *p* = 0.0004 (e) and 71% vs. 0%, *p* < 0.0001 (f)), and with tumor between 30 and 50 mm (51% vs. 0%, *p* < 0.0001 (g) and 30% vs. 0%, *p* = 0.0001 (h))

The results are summarized in Table 2. Patients treated with lobectomy had better outcomes than those treated with sublobar resection, with higher 5‐YSR (67% vs. 0%, *p* < 0.0001; Figure 2(a)) and 5‐YDFSR (65% vs. 0%, *p* < 0.0001; Figure 2(b)). When the extent of resection in relation to tumor size was evaluated, lobectomy was associated with a better 5‐YSRT and 5‐YDFSR in patients with tumor <20 mm (79% vs. 28%, *p* = 0.001, Figure 2(c), 89% vs. 38%, *p* = 0.001, Figure 2(d)), in patients with tumor between 20 and 30 mm (62% vs. 14.8%, *p* = 0.0004, Figure 2(e); 71% vs. 0%, *p* < 0.0001; Figure 2(f)) and in those with tumor between 30 and 50 mm (51% vs. 0%, *p* < 0.0001, Figure 2(g); 30% vs. 0%, *p* = 0.0001, Figure 2(h)).

### Survival in relation to immunoistochemical neuroendocrine markers

The results are summarized in Table 2. Immunoistochemical staining was positive for synaptophysin in 79 (67%) patients, for chromogranin A in 59 (50%) patients, and for NSE in 99 (84%). Sixty‐seven tumors (57%) were positive for all three neuroendocrine markers (triple‐positive group), while 50 (43%) were negative for one or two markers (nontriple‐positive group).

**FIGURE 3:**
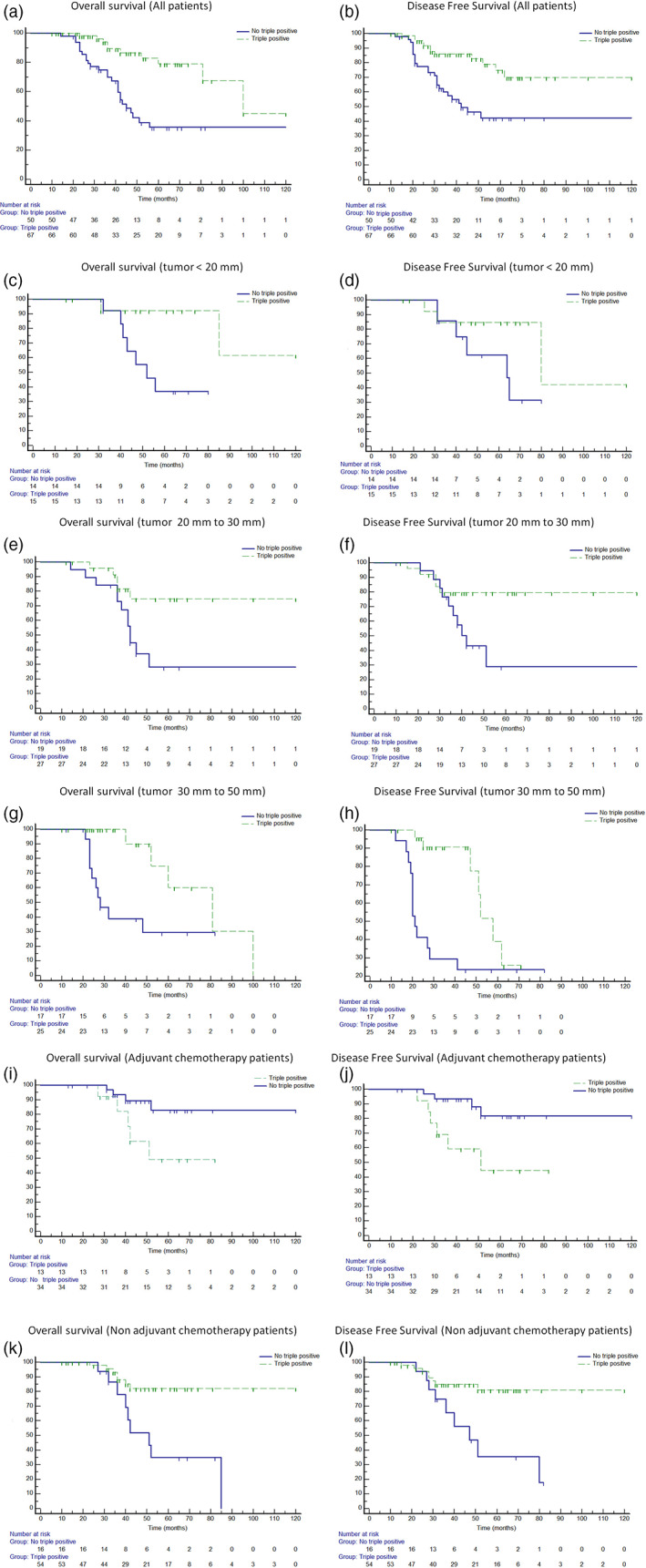
Five‐year survival and disease‐free survival rate in relation to immunoistochemical neuroendocrine expressions (triple vs. no triple‐positive groups) in all patients (79% vs. 35%, *p* = 0.0001 (a) and 69% vs. 42%, *p* = 0.0008 (b)), in patients with tumor <20 mm (92.3% vs. 36.9%, *p* = 0.01 (c) and 84% vs. 62%, *p* = 0.02 (d)), with tumor between 20 and 30 mm (74.9% vs. 28.1%, *p* = 0.01 (e) and 79% vs. 28%, *p* = 0.03 (f)), with tumor between 30 and 50 mm (60.4% vs. 29%, *p* = 0.0005 (g) and 38% vs. 23%, *p* = 0.003 (h)), in patients treated with adjuvant chemotherapy (61% vs. 90%, *p* = 0.043 (i) and 68% vs. 91%, *p* = 0.031 (j)), and with surgery alone (72% vs. 16%, *p* = 0.0003 (k) and 66% vs. 20%,*p* = 0.0008 (l))

5‐YSRT and 5‐DFSRT were better in the triple‐positive group compared to the nontriple‐positive group (79% vs. 35%, *p* = 0.0001, Figure 3(a); 69% vs. 42%, *p* = 0.0008 Figure 3(b)). These results were confirmed also in patients with tumor <20 mm (92.3% vs. 36.9%, *p* = 0.01, Figure 3(c); 84% vs. 62%, *p* = 0.02, Figure 3(d)), in patients with tumor between 20 and 30 mm (74.9% vs. 28.1%, *p* = 0.01, Figure 3(e); 79% vs. 28%, *p* = 0.03, Figure 3(f)) and in those with tumor between 30 and 50 mm (60.4% vs. 29%, *p* = 0.0005, Figure 3(g); 38% vs. 23%, *p* = 0.003, Figure 3(h)). When patients were stratified for the administration of adjuvant chemotherapy, in patients who received chemotherapy triple‐positive compared to no triple‐positive group had worse 5‐YSR (61% vs. 90%, *p* = 0.043, Figure 3(i); 5‐YDFSR (68% vs. 91%, *p* = 0.031, Figure 3(j)) while patients who did not receive chemotherapy triple‐positive compared to no triple‐positive group had better 5‐YSR (72% vs. 16%, *p* = 0.0003; Figure 3(k)) and 5‐YDFSR (66% vs. 20%, *p* = 0.0008; Figure 3(l)).

### Prognostic factors

The results are summarized in Table 3. Cox regression analysis showed that tumor size <20 mm, lobectomy, adjuvant chemotherapy, and triple‐positive immunistochemical neuroendocrine markers were significant favorable prognostic factors for overall survival and disease‐free survival while age, sex, comorbidity and histology did not affect overall survival and disease‐free survival.

**TABLE 3:** Prognostic factors for overall survival and disease‐free survival

Factors	Overall survival			Disease‐free survival		
	HR	95% CI	*p* value	HR	95% CI	*p* value
Age (≤70 vs. >70)	0.89	0.78–2.78	0.56	0.78	0.68–1.98	0.46
Sex (male vs. female)	1.07	0.97–1.87	0.76	1.17	0.87–1.37	0.66
Comorbidity (yes vs. no)	0.76	0.56–2.21	0.58	0.86	0.46–2.61	0.68
Tumor size (<20 vs. >20 mm)	2.98	1.45–2.98	0.001	2.38	1.25–2.65	0.002
Resection (lobar vs. sublobar)	4.19	2.21–3.34	0.002	4.45	2.31–4.54	0.001
Histology (pure vs. mixed)	1.56	1.98–4.91	0.49	1.34	1.54–3.87	0.51
Adjuvant chemotherapy	2.17	1.56–3.65	0.001	2.30	1.76–4.10	0.002
Triple positive markers	3.91	1.34–2.98	0.003	3.91	1.58–3.16	0.005

*Abbreviations*: CI, confidence interval; HR, hazard ratio.

## DISCUSSION

LCNEC has poor prognosis even in resected patients with early stage, and it is still debated whether this tumor should be treated in the same manner as NSCLC or SCLC. The topic of sublobar resection versus lobectomy for stage I tumors smaller than 20 mm is controversial in NSCLC,^5^, ^6^, ^7^ while lobectomy seems to be superior to sublobar resection even for early‐stage SCLC.^8^ Furthermore, adjuvant chemotherapy is indicated for stage II or III NSCLC^9^ while retrospective studies support the routine administration of adjuvant therapy even for stage I SCLC.^10^, ^11^, ^12^ Previous papers,^13^, ^14^, ^15^, ^16^, ^17^, ^18^, ^19^, ^20^, ^21^, ^22^, ^23^ summarized in Table 4, evaluated several prognostic factors as the extent of resection, the administration of adjuvant chemotherapy, and the expression of immunoistochemical neuroendocrine markers to define the best treatment of LCNEC in relation to its clinical and pathological characteristics. However, the results were contrast as the exisisting studies were heterogenous. Some studies evaluated all stages of LCNEC^16^, ^19^, ^20^, ^21^, ^22^ while others included only patients with early stage.^13^, ^14^, ^15^, ^17^, ^18^, ^23^ Some studies^16^, ^19^ included patients undergoing different perioperative treatment as radiotherapy and chemotherapy administered before and after operation while others evaluated only patients who received adjuvant chemotherapy alone^14^, ^17^, ^18^, ^20^, ^21^, ^22^, ^23^ or associated with radiotherapy.^13^, ^15^ Yet, only a few studies evaluated the extent of resections,^13^, ^14^, ^15^ but none of these correlated the type of resection with the expression of immunoistochemical neuroendocrine markers, as in the present. To overcome these limitations, the present study included only patients with early‐stage LCNEC who received adjuvant chemotherapy alone as radiotherapy has been found to have detrimental effects on survival. As previously reported by Wakeam et al.,^13^ also in this study the subgroups of patients were divided based on tumor size rather than T stage as this may be more applicable to clinical practice given the significative changes of lung cancer staging editions occurred within the study period (10 years). Furthermore, for the first time we evaluated in the same population all prognostic factors, such as adjuvant chemotherapy, extent of resection, and immunoistochemical neuroendocrine expression, to stratify the best treatment for each subgroup of patients.

**TABLE 4:** Literature review

Authors	Population	Variables	Results	Conclusions
Wakeam et al.^13^	1770 resected pts Surgery alone 1.307 pts Surgery + ACT 463 pts	5‐YSR (ACT vs. NACT) Prognostic Factors	59% vs. 45%, *p* < 0.0001, all pts 59.8% vs. 42%, *p* < 0.0001, >3 cm 60% vs. 42%, *p* = 0.002, 2–3 cm 54% vs. 51%, *p* = 0.27, <2 cm ACT, *p* < 0.0001 T stage *p* = 0.006 R1 *p* = 0.008 Sublobar resection *p* < 0.0001 CT within 3 months *p* < 0.0001, within 3–6 months *p* = 0.005	ACT was associated with significantly longer survival for tumors larger than 3 cm and possibly for tumors between 2 and 3 cm
Kujtan et al.^14^	1232 pts Surgery alone 957 (77.7%) Surgery + ACT 275 (22.3%)	5‐YSR (ACT vs. NACT) Prognostic factors	64% vs. 48%, *p* < 0.001, all pts 59% vs. 50%, *p* = 0.006, stage IA 68% vs. 44% *p* < 0.001, stage IB Age < 70 y, *p* < 0.0001 Non white, *p* = 0.002 Lobectomy, *p* = 0.0003 ACT, *p* < 0.0001	ACT improved survival in patients with stage IA and stage IB
Kim et al.^15^	139 pts Surgery alone 50 pts Surgery + AT (CT and/or CT + RT) 89 pts	5‐YSR (AT vs. NAT) 5‐YDFSR (AT vs. NAT) Prognostic factors 5‐YSR 5‐YDFSR	62% vs. 48%, *p* = 0.212, all pts 100% vs. 61%, *p* = 0.2, stage I 52% vs. 31%, *p* = 0.02, stage II 46% vs. 35%, *p* = 0.308, all pts 80% vs. 50%, *p* = 0.3, stage I) 39% vs. 18%, *p* = 0.03, stage II pN (*p* < 0.001) R0 resection (*p* = 0.02) AT (*p* = 0.003) pN (*p* < 0.001) Pneumonectomy (*p* = 0.04) R0 resection (*p* = 0.009) AT (*p* < 0.001)	AT improved survival in patients with stage II or higher
Veronesi et al.^16^	144 resected pts 21 had induction therapy and 24 ACT	5‐YSR 3‐YSR (ACT vs. NACT) Prognostic factors	43% all pts, 52% stage I, 59% stage II, 20% stage III 100% vs. 58%, *p* = 0.077 Pneumonectomy, *p* = 0.02 Stage III, *p* = 0.004	There is a trend to better outcome with chemotherapy in stage I disease
Tanaka et al.^17^	63 resected pts Surgery alone 40 pts Surgery + ACT 23 pts	5‐YSRT (ACT vs. NACT)	74.4% vs. 32.3%, *p* = 0.042	There is a trend to better outcome with chemotherapy in stage I disease
Raman et al.^18^	2642 pts Surgery alone 2.161 pts Surgery + ACT 481 pts	5‐YSR (ACT vs. NACT) Prognostic factors IA, IB	53%, all pts 56% vs. 54%, *p* = 0.1, stage IA 62% vs. 43%, *p* < 0.0001, stage IB Lobectomy, *p* < 0.001 Lobectomy, *p* = 0.02 ACT, *p* = 0.007	ACT improved survival in stage IB but not in stage IA
Roesel et al.^19^	251 pts Surgery alone 150 pts Surgery + ACT 101 pts (19 had induction therapy)	5‐YSR (ACT vs. NACT)	60.9% stage I, 31% stage II, 22% stage III 34.6% vs. 37.8%, *p* = 0.02 for all pts, *p* = 0.005 for stage I, *p* = 0.001 for stage II	ACT may improve survival in stage Ib and higher
Sarkaria et al.^20^	100 resected pts 24 had induction therapy and 20 of these ACT	5‐YSR (ACT vs. NACT) Prognostic factors	50% vs. 45%, *p* = 0.1, all pts 37% vs. 51%, *p* = 0.052, stage IB‐IIIA Gender (*p* = 0.007) Co‐morbidity (*p* = 0.012) Stage (*p* = 0.011)	ACT may improve survival in advanced‐stage patients
Iyoda et al.^21^	72 resected pts Surgery alone 42 Surgery + ACT 30 pts	Recurrence (ACT vs. NACT) 5‐YDFSR (ACT vs. NACT) Prognostic factors for 5‐YDFSR	10 (33%) vs. 26 (61.9%) (*p* = 0.017) 58.9% vs. 33%, *p* = 0.044 ACT, *p* = 0.005 Stage, *p* = 0.025 Second cancer, *p* = 0.008	ACT is useful to prevent recurrence
Iyoda et al.^22^	38 resected pts Surgery alone 23 pts Surgery + ACT 15 pts	2‐YSR (ACT vs. NACT) 5‐YSR (ACT vs. NACT) 2‐YDFSR (ACT vs. NACT) 5‐YDFSR (ACT vs. NACT)	88.9% vs. 65.2%, *p* = 0.025 88.9% vs. 47.4%, 86.7% vs. 47.8%, *p* = 0.013 86.7% vs. 34.8%	ACT was associated with significantly longer survival
Saji et al.^23^	45 pts Surgery alone 22 pts Surgery + ACT 23 pts	5‐YSR (ACT vs. NACT) Prognostic factors for 5‐YSR	87.5% vs. 58.5%, *p* = 0.04 ACT, *p* = 0.045	Adjuvant chemotherapy improved the survival even in stage I disease

*Abbreviations*: 5‐YSR, 5‐year survival rate; 5‐YDFSR, 5‐year disease free survival rate; ACT, adjuvant chemotherapy; AT, adjuvant therapy; pts, patients.

The clinico‐pathologic features of our study population were similar to other studies.^13^, ^14^, ^15^, ^16^, ^17^, ^18^, ^19^, ^20^, ^21^, ^22^, ^23^ LCNECs generally affected males (74%) and almost exclusively smokers (94%). CT scan did not presented specific features that allowed LCNEC to be differentiated from other NSCLCs and preoperative diagnosis of LCNEC was obtained in only 9% of cases while in most cases (91%) the diagnosis of LCNEC was obtained by careful identification of cell morphology, mitotic phase and immunohistochemical markers of surgical specimens.

First, in line with other previous papers, adjuvant chemotherapy was associated with better survival outcomes compared to surgery alone. This survival association was found for patients with tumors >20 mm, and was strongest for those with tumors >30 mm, but no significant differences were found for patients with tumor <20 mm. Our results were in contrast with those of Kujtan et al.,^14^ who found that adjuvant chemotherapy was associated with a better survival in both stage IA and IB patients. The benefit remained significant after multivariable adjustment and was further supported by propensity score‐matched analyses. In this analysis, the tumor stage was classified based on the International Union Against Cancer/American Joint Committee on Cancer (UICC/AJCC) TNM staging system sixth and seventh edition staging classification, which limited the comparison with our data. Furthermore, other authors did not find any advantages associated with the administration of adjuvant chemotherapy for stage I patients. Kim et al.^15^ found significant survival benefit from adjuvant treatment only for stage II or higher, but not for stage I. However, 30% of patients with stage I LCNEC presented distant recurrence independently whether they received adjuvant therapy or not. Yet, multivariate analysis showed that adjuvant therapy was a significant survival prognostic factor. All these factors may likely demonstrate the benefit of adjuvant therapy for LCNEC also in early stage. Veronesi et al.^16^ and Tanaka et al.^17^ found a significant survival benefit for adjuvant chemotherapy in the whole population, but it was not significant for stage I disease. However, in both papers there was a trend to better outcome with chemotherapy in stage I disease, and probably the small number of subjects did not allow a statistically significant difference to be obtained.

Second, lobectomy was associated with better survival not only for patients with large tumors (>30 mm) but also in those with small lesions (<30 and <20 mm). Yet, lobectomy was a favorable prognostic factor for survival in multivariate analysis, in line with previous studies. In a large retrospective study including 1530 patients with all‐stage LCNEC, Cao et al.^24^ found that surgery was a positive independent prognostic factor for survival, and lobectomy was associated with better outcomes compared to other types of resections, such as sublobar or pneumonectomy. Similarly, Wakeam et al.^13^ reported that sublobar resection for stage I LCNEC was correlated with worse survival than lobectomy. Iyoda et al.^22^ found that patients with limited resection of primary LCNEC tumors experienced more recurrence than those undergoing lobectomy.

Third, patients with triple‐positive markers had better survival outcomes than the control group and these results were also observed when patients were stratified according to tumor size. Neuroendocrine markers are often negative in poorly differentiated neuroendocrine cancers. Thus, LCNEC with nontriple‐positive markers tended to be similar to SCLC and thus associated with a poor prognosis. By contrast, in patients receiving adjuvant chemotherapy, triple‐positive patients had worse survival compared to nontriple‐positive patients. As observed by Tanaka et al.,^17^ the LCNEC might become resistant to chemotherapy through coexistence and mutual interaction of all three proteins while the lack of any of these proteins may reduce the resistance of tumor to chemotherapy. Similarly, SCLCs that show a poor prognosis have a high initial response rate to chemotherapy.

**FIGURE 4:**
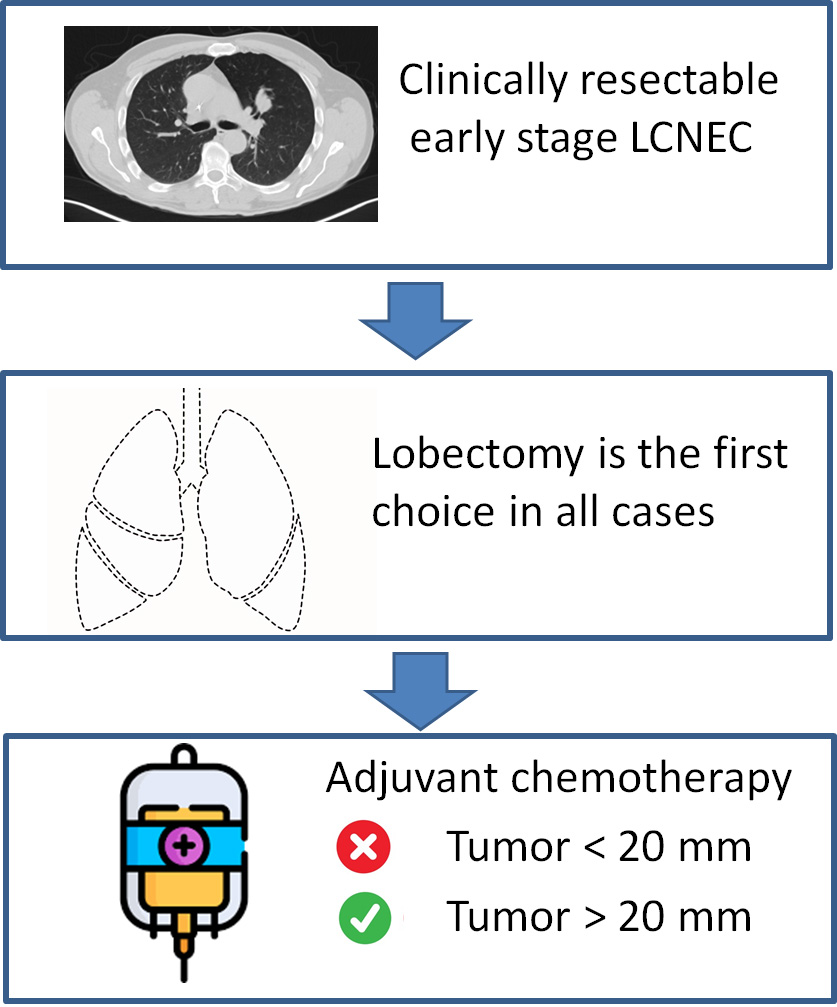
Therapeutic strategy in patients with early‐stage large cell neuroendocrine carcinoma

Fourth, from a clinical point view the results of this study suggest different treatments in relation to characteristics of LCNEC, as summarized in Figure 4. Patients with LCNEC scheduled for surgical resection should be treated in a similar way as for SCLC, and lobectomy routinely performed also even for small tumors (<20 mm). By contrast, adjuvant chemotherapy should be not routinely administered in patients with LCNEC as performed for SCLC, but only in patients with tumor >20 mm. This is in line with the current National Comprehensive Cancer Network (NCCN) guidelines^25^ that recommend adjuvant therapy in patients with “high‐risk” features, including poorly differentiated neuroendocrine histology and pathologic stage IB NSCLC, but do not explicitly recommend routine adjuvant therapy for stage IA and IB LCNEC. The lack of triple‐positive markers seems to be associated with poor prognosis, but a better response to chemotherapy. In theory, it may influence the decision of adjuvant chemotherapy in selected patients with tumor size <20 mm (i.e. nontriple‐positive markers). However, our data are not strong enough to support that different neuroendocrine marker profiles may influence therapeutic strategy. Future studies, including molecular studies, may improve the treatment stratification of these subsets of patients. Rossi et al.^26^ analyzed the molecular profile of 83 LCNEC patients. They found that patients with mesenchymal epithelial transition factor (MET)‐positive samples had better median overall survival than the control group with MET‐negative samples (24 vs. 18 months). Other authors^27^, ^28^ supported the use of epidermal growth factor receptor (EGFR)‐targeted therapy due to the presence of EGFR‐activating mutations in mixed LCNECs with an adenocarcinoma component, while Mairinger et al.^29^ hypothized the use of anti‐angiogenetic‐targeted drugs in association with chemotherapy as the angiogenesis could be involved in LCNEC metastasization. Furthermore, other innovative therapeutic targets could be represented by tropomyosin‐related kinase B and brain‐derived neurotrophic factor, which are highly expressed in LCNEC.^30^

## STUDY LIMITATIONS

This study had some limitations that should be considered before drawing definitive conclusions. First, because of the retrospective and multicenter nature of the study, the choice of type of resection (lobectomy or sublobar), multimodality treatment (surgery plus chemotherapy or surgery alone), adjuvant chemotherapy regimen (SCLC‐based regimen or NSCLC‐based regimens), dosages and timing of administration of chemotherapy, and the strategy for management of recurrence (CT, RT, combined CT and RT) was based on the decision of each participating center rather than on structured protocol. Second, patients who received adjuvant chemotherapy may have been selected among those with better functional status, thus the effect attributed to treatment could be due to patients' more favorable status. Third, the sublobar group included patients undergoing segmentectomy and wedge resection. However, anatomic segmentectomy has traditionally been considered superior to wedge resection and this could be affect the results. Due to the relative rarity of LCNEC, the study population was rather small, precluding the ability to obtain more powerful results.

## CONCLUSIONS

LCNEC represents a rare entity of neuroendocrine pulmonary malignancies that is associated with poor prognosis and high recurrence rate, also in patients with early stage cancer undergoing surgical resection. Lobectomy should be routinely performed for management of limited LCNEC while adjuvant chemotherapy is indicated in patients with tumor >20 mm. The presence of multiple immunoistochemical neuroendocrine markers is also associated with a poor prognosis in early‐stage LCNEC. Because of the small sample size in this paper, a multicenter, prospective, randomized control trial is necessary to define the role of adjuvant chemotherapy in early‐stage LCNEC in relation to immunoistochemical neuroendocrine expressions.

## CONFLICT OF INTEREST

The authors declare that they have no conflict of interest.
